# Retraction: Comprehensive circular RNA expression profile of lung adenocarcinoma with bone metastasis: identification of potential biomarkers

**DOI:** 10.3389/fgene.2025.1637633

**Published:** 2025-06-09

**Authors:** Frontiers Editorial Office

The journal retracts the 16 August 2022 article cited above.

Following publication, concerns were raised regarding the integrity of the images in the published figures. The authors failed to provide a satisfactory explanation during the investigation, which was conducted in accordance with Frontiers’ policies.

This retraction was approved by the Chief Executive Editor of Frontiers. The authors agree to this retraction.

